# Artesunate Induces ROS-Mediated Apoptosis in Doxorubicin-Resistant T Leukemia Cells

**DOI:** 10.1371/journal.pone.0000693

**Published:** 2007-08-01

**Authors:** Thomas Efferth, Marco Giaisi, Annette Merling, Peter H. Krammer, Min Li-Weber

**Affiliations:** 1 Pharmaceutical Biology of Natural Products, German Cancer Research Center, Heidelberg, Germany; 2 Tumor Immunology Program, German Cancer Research Center, Heidelberg, Germany; Dresden University of Technology, Germany

## Abstract

**Background:**

A major obstacle for successful cancer treatment often is the development of drug resistance in cancer cells during chemotherapy. Therefore, there is an urgent need for novel drugs with improved efficacy against tumor cells and with less toxicity on normal cells. Artesunate (ART), a powerful anti-malarial herbal compound, has been shown to inhibit growth of various tumor cell lines *in vitro* and of xenografted Kaposi's sarcoma in mice *in vivo*. However, the molecular mechanisms by which ART exerts its cytotoxicity have not been elucidated. The ART-class of anti-malarial compounds is attractive due to their activity against multidrug-resistant *Plasmodium falciparum* and *Plasmodium vivax* strains. Another salient feature of these compounds is the lack of severe side effects in malaria patients.

**Methodology and Principal Findings:**

In this study, we used T-cell leukemias as a model system to study the molecular mechanisms of ART-induced apoptosis. The most typical anticancer drugs are DNA intercalators such as Doxorubicin. To investigate drug sensitivity and resistance, we chose a Doxorubicin-resistant leukemia cell line and investigated the killing effect of ART on these cells. We show that ART induces apoptosis in leukemic T cells mainly through the mitochondrial pathway *via* generation of reactive oxygen species (ROS), a mechanism different from Doxorubicin. This is confirmed by the fact that the antioxidant N-Acetyle-Cysteine (NAC) could completely block ROS generation and, consequently, inhibited ART-induced apoptosis. Therefore, ART can overcome the Doxorubicin-resistance and induce the Doxorubicin-resistant leukemia cells to undergo apoptosis. We also show that ART can synergize with Doxorubicin to enhance apoptotic cell death in leukemic T cells. This synergistic effect can be largely explained by the fact that ART and Doxorubicin use different killing mechanisms.

**Conclusions:**

Our studies raise the possibility to develop ART in combination with other established anticancer drugs which induce apoptosis through the pathways or mechanisms different from ART.

## Introduction

Half a century after launching chemotherapy for tumor treatment [1], antineoplastic drugs have become an indispensable part of the armory to combat hematopoietic malignancies. Marine and terrestrial plants are particularly suitable to find novel drugs with anti-tumor activity. Prominent examples for the success of natural products originally obtained from plants to fight cancer are the *Vinca* alkaloids from *Catharanthus roseus*, the DNA topoisomerase I inhibitor camptothecin from *Camptotheca acuminata*, the terpene paclitaxel from *Taxus baccata*, and the lignan podophyllotoxin, isolated from *Podophyllum peltatum* [2]. In fact, 69% of anti-cancer drugs approved between 1940 and 2002 are either natural products or developed on the basis of knowledge gained from natural products. About ¾ of plant-derived drugs used today in the clinic come from traditional medicines [3], [4].

Artemisinin (ARS) also known as *qinhaosu*, is the active principle of the Chinese plant *Artemisia annua* L. (*qinhao*) used for more than 2000 years in Traditional Chinese Medicine (TCM) as a remedy to treat fever and chills caused by malaria infections [5]. A program for the discovery of new anti-malarial drugs from TCM launched by the Chinese government in 1972 led to the identification of ARS [6]. ARS is a sesquiterpene lactone containing an endoperoxide bridge representing the active moiety of the molecule. ARS exert its anti-malarial activity by generation of organic free radicals through cleavage of the endoperoxide bridge. The radical molecules cause macromolecular damage by alkylating and poisoning one or several essential malarial proteins [7].

Due to the low solubility of ARS in oil and water, several semisynthetic derivatives have been developed, including artemether, arteether, artesunate (ART) and others. ART was found to be the most potent derivative *in vitro* [8]. The ARS-class of anti-malarial compounds is attractive due to their activity against multidrug-resistant *Plasmodium falciparum* and *Plasmodium vivax* strains [9]. Another salient feature of these compounds is the lack of severe side effects in malaria patients [10], [11]. Neurotoxicity, however, occurs in animals after prolonged treatment with supra-therapeutic doses [12], [13].

Over the last 10 years, studies have shown that ARS and ART also possess profound cytotoxicity against tumor cells in *vitro* and in *vivo* [14]–[18]. We have previously shown that ART triggers apoptotic cell death in various tumor cell lines in both a p53-dependent and –independent manner [19], [20]. Apoptosis involves two main pathways: the extrinsic pathway, initiated by binding of ligands to specific death receptors, and the intrinsic pathway, initiated at the mitochondria [21], [22]. Triggering the extrinsic apoptosis pathway leads to the formation of the death inducing signaling complex (DISC) containing the FAS-associated death domain adaptor protein FADD and pro-caspase-8 and -10. Activated caspase-8 cleaves and activates the effector caspase-3, and activated caspase-3 cleaves target “death proteins” such as poly (ADP-ribose) polymerase (PARP) leading to apoptosis. Intrinsic death stimuli directly or indirectly activate the mitochondrial pathway by inducing the release of cytochrome c from the mitochondria and the formation of the apoptosome complex with Apaf-1 and pro-caspase-9 in the cytoplasm. Caspase-9 is activated at the apoptosome and, in turn, activates caspase-3 and cleaves PARP [21], [22]. This death pathway is largely controlled by the pro-apoptotic (*e.g.* Bax, Bad, Bid and Bak) and anti-apoptotic (*e.g.* Bcl-2 and Bcl-x_L_) Bcl-2 family proteins [21], [23].

So far, the molecular mechanisms by which ART induces apoptosis in tumor cells have not been elucidated. Therefore, the aim of this study was to analyze the precise apoptotic pathways induced by ART. In addition, tumors may develop resistance to a particular drug during chemotherapy. Therefore, it would be interesting to know, whether ART could overcome drug-resistance of tumors and if so, by what mechanisms. To investigate these questions we chose leukemic T cells as a model system. We show that ART induces apoptosis *via* the intrinsic death pathway in all leukemic T-cell lines tested. To investigate drug sensitivity and resistance, we chose a Doxorubicin-resistant cell line and showed that ART induced apoptotic cell death in these cells. Our study demonstrated that ART induces apoptosis through a mechanism different from Doxorubicin and, thus, overcomes the drug-resistance of the tumor cells. Furthermore, we show that ART synergistically enhances Doxorubicin-induced apoptosis due to using different killing mechanisms.

## Materials and Methods

### Cell lines and culture

The human acute T cell leukemia Jurkat cell line J16, the human acute lymphoblastic leukemia cell lines CEM and Molt-4, the human T cell lymphoma Hut78, the genetically engineered cell lines J-Neo, J-Bcl-2, J-caspase-8^−/−^, Jurkat A3 FADD^−/−^ and the parental Jurkat A3 were cultured in RPMI 1640 medium (GIBCO laboratories, Grand Island, NY) supplemented with 10% FCS, 50 µg/ml Gentamicin (GIBCO), 10 mM HEPES (GIBCO, 1 M solution), and 2 mM L-glutamine (GIBCO, 200 mM solution) at 37°C and 5% CO_2_. All cell lines were purchased from American Type Culture Collection (ATCC, Manassas, USA). Generation of the Doxorubicin-resistant cell line CEM-Dox_R_ (CCRF-CEM/ADR5000) was described previously [24]. The multi-drug resistance phenotype of this cell line has been described previously [19], [25].

### Determination of apoptosis

Cells were plated in triplicates and treated for the indicated periods of time at 37°C with different drugs. The pan-caspase inhibitor zVAD-fmk was purchased from Bachem (Weil am Rhein, Germany). Apoptotic cell death was examined by two parameters: FSC/SSC index of apoptotic-like change in cell size and granularity by FACScan and by analysis of DNA fragmentation according to the method of Nicoletti [26]. Briefly, cells were resuspended in a buffer containing 0.1% (w/v) sodium citrate, 0.1% (v/v) Triton X-100 and 50 µg/ml propidium iodide (Sigma). After incubation at 4°C in the dark for at least 16 h apoptotic nuclei were quantified by FACScan (Becton Dickinson).

### Western blot analysis

1×10^6^ cells were sedimented and lysed for 15 min in ice-cold lyses buffer (15 mM Tris-HCl, pH 7.4, 137 mM NaCl, 10% (w/v) Glycerin, 1% (v/v) Triton X-100, 2 mM EDTA, 1 mM PMSF, 0.4 mM Na_3_VO_4_, 10 mM NaF, complete protease inhibitor cocktail, Roche). After removing the cell debris by centrifugation at 13,000 rpm for 15 min, equal amounts of proteins were seperated on a 12% SDS-PAGE under reducing conditions, blotted onto a nitrocellulose membrane (Amersham Biosciences, Little Chalfon, UK) and blocked with 5% non-fat drymilk in PBS/Tween (0.05% Tween-20 in PBS). The following antibodies were used: caspase-2 mAb (mouse IgG1, Cell Signaling, Germany), Cytochrome c (Cell Signaling), caspase-9 mAb (Santa Cruz Biotechnology, Santa Cruz, CA), caspase-3 polyclonal antibody (Cell Signalling), caspase-8 mAb C15 (mouse IgG2b) recognizes the p18 subunit of caspase-8 (made in our lab) [27], PARP [poly (ADP-ribose) polymerase] mAb (BD PharMingen, Germany) and tubulin (Sigma, Taufkirchen, Germany). Enhanced chemiluminescence (PerkinElmer Life Sciences, Boston, MA) was used for detection. For stripping, blots were incubated for 30 min in a buffer containing 62.5 mM Tris/HCl, pH 6.8, 2% SDS, and 100 mM β-mercaptoethanol at 56°C. The blots were washed six times for 10 min in PBS/Tween and blocked again in 5% non-fat drymilk.

### Redox measurement

Doxorubicin (500 ng/ml) or ART (2–4 µg/ml) treated cells were stained for 30 min with 5 µM of the H_2_O_2_-sensitive fluoresent dye dichlorofluorescein diacetate (DCFDA, FL-1) (Molecular Probes, Inc., Eugene, OR) at 37°C in the dark, washed 3 times with PBS and subsequently assayed by FACScan.

## Results

### ART induces apoptosis in leukemic T cells

**Figure 1:**
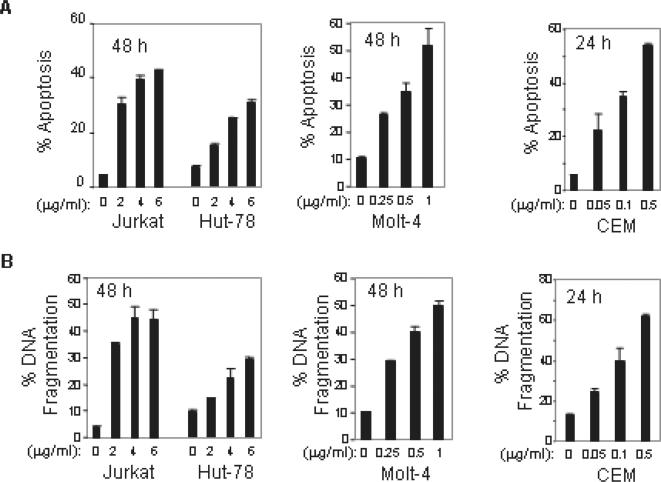
ART induces apoptosis in malignant T cells. Jurkat, Hut-78, Molt-4 and CEM leukemic T cells were incubated with different doses of ART for 24 or 48 h as indicated. The apoptotic cell death was determined either by FSC/SSC index for changes in cell size and granularity (A) or by DNA fragmentation (B). Results are representative of three independent experiments determined in triplicates.

To investigate the cytotoxicity of ART in T cell leukemias, four different leukemic T cell lines CCRF-CEM, Jurkat, Hut-78, and Molt-4 were subjected to ART treatment. Apoptotic cell death was examined by two parameters: FSC/SSC index of apoptotic-like changes in cell size and granularity (Fig. 1A), and DNA fragmentation (Fig. 1B). Treatment with ART resulted in a dose-dependent induction of apoptotic cell death in all malignant T cell lines tested. The minimum ART concentration to induce 30–50% of Jurkat, Hut-78 and Molt-4 cells to undergo apoptosis in 48 h was about 2, 6 and 0.5 µg/ml, respectively. The cytotoxic effect of ART on CCRF-CEM cells was even stronger. The minimum concentration of ART to induce 30–50% apoptosis in CEM cells in 24 h was about 0.1 µg/ml. The data demonstrate that ART can induce substantial and variable apoptotic cell death in different leukemic cells.

### ART induces apoptosis through the intrinsic pathway

**Figure 2:**
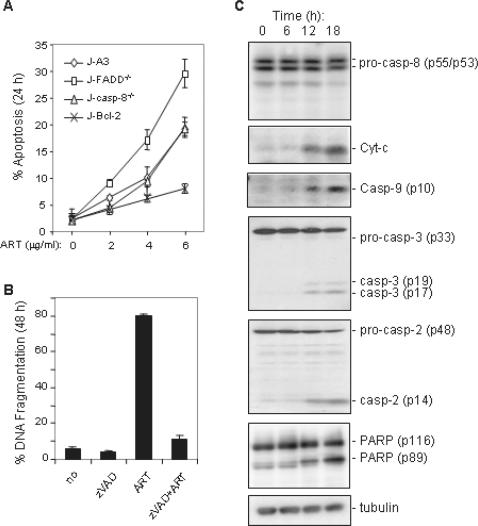
ART induces apoptosis through the intrinsic (mitochondria) pathway. (A) The death receptor system is not required for ART-induced apoptosis. Jurkat cells deficient in FADD (FADD^−/−^), caspas-8 (casp-8^−/−^), or over-expressing Bcl-2 and parental (A3) Jurkat cells were treated with different does of ART. Apoptotic cell death was determined by FSC/SSC in triplicates. (B) ART-induced apoptosis involves caspases. Jurkat cells were treated with ART (4 µg/ml) in the presence or absence of 50 µM of pan-caspase inhibitor zVAD-fmk for 48 h. Apoptotic cell death was determined by DNA fragmentation in triplicates. (C) ART induces cytochrome c release and activation of caspase-2, 3 and 9. CEM leukemia cells were treated with 1 µg/ml ART for different times as indicated. Cell lysates were subjected to Western blotting with antibodies against cytochrome c, caspase-2, 3, 8, 9, PARP, and control antibodies against tubulin. Data are representative of three independent experiments.

Since apoptotic cell death may be triggered through the extrinsic (receptor-mediated) or the intrinsic (mitochondria-mediated) pathway, we asked which death pathway was involved in ART-induced cell death. To investigate this question, Jurkat T cells deficient of either the death adapter molecule FADD (J-FADD^−/−^) or caspase-8 (J-casp-8^−/−^) were treated with different concentrations of ART. The experiment showed that both J-FADD^−/−^ and J-casp-8^−/− ^cells were at least as sensitive to ART as the parental J-A3 cells indicating that the extrinsic pathway was not involved in ART-mediated apoptosis (Fig. 2A). This assumption was supported by a further experiment showing that Jurkat T cells stably expressing the anti-apoptotic protein Bcl-2 (J-Bcl-2) were significantly resistant to ART-induced apoptosis (Fig. 2A). ART-induced apoptosis were completely blocked by the broad caspase inhibitor zVAD demonstrating that caspases were involved in the death process (Fig. 2B). Kinetic analysis of proteins involved in the apoptotic signaling pathway showed that ART did not induce activation of caspase-8, the main caspase of the extrinsic pathway (Fig. 2C). Instead, release of cytochrome c and activation of caspase-9, the main caspase involved in the intrinsic pathway, were observed upon ART treatment (Fig. 2C). ART treatment also induced activation of caspase-2 (Fig. 2C). These data demonstrate that ART induces apoptosis through the intrinsic pathway.

### ART-induces apoptosis by generation of ROS

**Figure 3:**
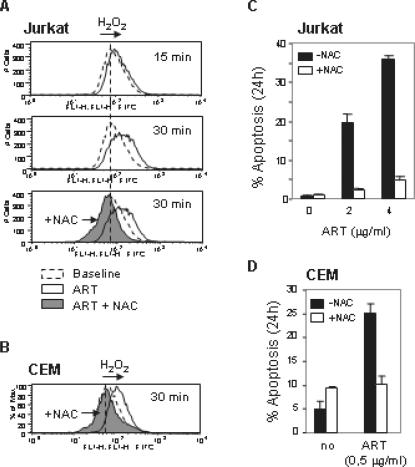
ROS mediate ART-induced apoptosis in leukemia cells. (A) Jurkat cells were treated with 4 µg/ml of ART for different times as indicated and the redox status was monitored by the oxidation-sensitive fluorescent dyes for H_2_O_2_. (B) CEM cells were treated with 0.5 µg/ml doses ART. After 30 min, the redox status was measured as in (A). (C) and (D) ART-induced apoptosis was blocked by the antioxidant NAC. Jurkat (C) and CEM (D) cells were treated with ART in the presence (15 mM) or absence of NAC for 24 h. Apoptotic cell death was analyzed by FSC/SSC in triplicates. Results are representative of four independent experiments.

The intrinsic pathway can be triggered by many stimuli including ROS. Mitochondria are the major site of ROS production, and accumulation of ROS may lead to the initiation of apoptosis [28]. Several studies imply that inhibition of apoptosis by Bcl-2 is associated with protection against ROS [29], [30]. ARS has been shown to exert its anti-malarial activity by generation of organic free radicals through cleavage of the endoperoxide bridges [7]. To investigate whether ART killed tumor cells by inducing generation of ROS, we monitored the redox status using the oxidation-sensitive fluorescent dye, DCFDA, in Jurkat and CEM T cells. We found that ART induced a rapid accumulation of H_2_O_2_ (as early as 15 min) in Jurkat T cells which were completely blocked by the anti-oxidant N-Acetyle-Cysteine (NAC) (Fig. 3A). Similar results were observed in CEM cells (Fig. 3B). To further investigate whether ART-induced ROS is required for induction of apoptosis, Jurkat and CEM T cells were treated with ART in the presence or absence of NAC. The experiments showed that NAC almost completely blocked ART-induced apoptosis demonstrating that ROS production is the cause of ART-mediated apoptotic cell death in leukemic T cells (Fig. 3C and D).

### ART induces Doxorubicin-resistant leukemic T cell to undergo apoptosis

**Figure 4:**
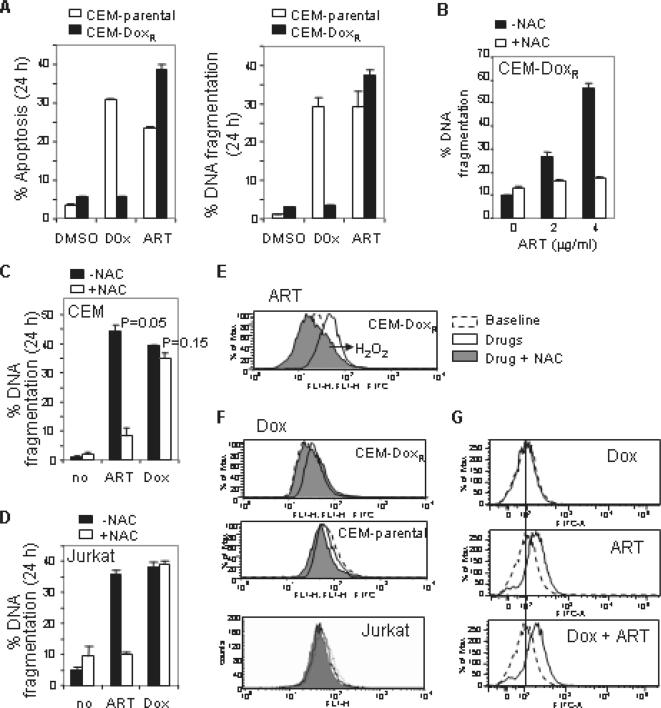
ART induces Doxorubicin-resistant leukemic cells to undergo apoptosis by a mechanism different from the one induced by Doxorubicin. (A) ART induces apoptosis in Doxorubicin-resistant leukemic cells. Doxorubicin-resistant (CEM-Dox_R_) and parental (CEM-parental) CEM cells were treated with either 1 µg/ml of ART or 0.5 µg/ml of Doxorubicin for 24 h. Apoptosis was determined by FSC/SSC (left panel) and DNA fragmentation (right panel). Results are representative of two independent experiments. (B) NAC inhibits ART-induced apoptosis in Doxorubicin-resistant leukemic cells. CEM-Dox_R_ cells were treated with different doses of ART in the presence or absence of NAC (15 mM). Apoptotic cell death was determined by DNA fragmentation in triplicates. (C) and (D) NAC does not inhibit Doxorubicin-induced apoptosis. CEM-parental (C) and Jurkat (D) cells were treated either with ART or Doxorubicin in the presence or absence of NAC. Apoptotic cell death was determined by DNA fragmentation. Results are representative of four independent experiments. The p value was determined by the statistic program of Microsoft Excel. (E) ART induces ROS generation in CEM-Dox_R_ cells. CEM-Dox_R_ cells were treated with 4 µg/ml of ART in the presence or absence of NAC (15 mM) for 30 min. The redox status was measured as in figure 3. (F) Doxorubicin does not induce ROS in leukemic T cells. CEM-Dox_R_, CEM-parental and Jurkat cells were treated with 0.5 µg/ml of Doxorubicin in the presence or absence of NAC for 30 min. The redox status was measured as in (E). Results are representative of three independent experiments. (G) Doxorubicin does not interfere with the redox assay. CEM cells were treated with Doxorubicin (2 µg/ml) or ART (4 µg/ml) alone or in combination for 30 min. The redox status was measured as in F.

One of the main problems of present chemotherapy is the development of resistance of tumor cells towards drug-induced apoptosis. Therefore, there is a quest for the improvement of anticancer drugs. The most typical anticancer drugs are DNA intercalators such as Doxorubicin. To investigate whether ART could overcome such resistance, a Doxorubicin-resistant CEM sub-cell line CEM/ADR5000 [24] was treated with ART. As shown in Fig. 4A, CEM/ADR5000 (CEM-Dox_R_) cells were resistant whereas the parental cells CCRF-CEM (CEM-parental) were sensitive to Doxorubicin treatment. The experiment showed that ART was capable to induce apoptosis in CEM-Dox_R_ cells determined by both FSC/SSC (Fig. 4A, left panel) and DNA fragmentation (Fig. 4A, right panel). Similar to other leukemic T cells, ART-induced apoptosis of CEM-Dox_r_ cells could be blocked by NAC (Fig. 4B). In contrast, NAC could not block Doxorubicin-induced apoptosis in CEM (Fig. 4C) and Jurkat T cells (Fig. 4D). Further investigation of the ROS status after ART or Doxorubicin treatment revealed that accumulation of ROS was observed in ART treated CEM-Dox_R_ cells and the ART-induced ROS production was blocked by NAC (Fig. 4E). In contrast, Doxorubicin at the doses that induce apoptosis in CEM and Jurkat T cells did not show induction of ROS production (Fig. 4F). The absence of ROS production in Doxorubicin treated cells is not due to the intrinsic fluorescence of Doxorubicin that interferes with the assay for ROS. As shown in Fig. 4G, equal levels of ROS were detected when cells were treated with ART in the absence or presence of Doxorubicin. Thus, ROS is not involved in Doxorubicin-induced apoptosis in these cells and ROS is not involved in Doxorubicin-induced apoptosis in these cells.

### ART synergizes with Doxorubicin to enhance apoptosis

**Figure 5:**
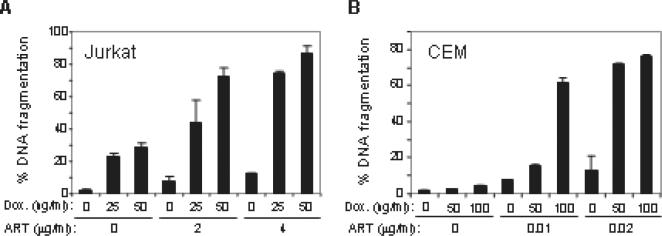
ART sensitizes malignant T cells to Doxorubicin-induced apoptosis. Jurkat (A) and CEM (B) cells were treated with combinations of different doses of ART and Doxorubicin for 24 h as indicated. The drugs were added at the same time. Apoptotic cell death was quantified by DNA fragmentation in triplicates. Results are representative of three independent experiments. The p value was determined by a two-way ANOVA test with factors Dox-, ART-, and their interaction. For Jurkat cells, the p values are p<0.0001 for Dox- and ART-treatment and p = 0.0006 for the combination treatment. For CEM cells, the p values are p<0.0001 for Dox-, ART- as well as the combination treatment.

Since ART induces apoptosis by a mechanism different from that of Doxorubicin, we speculated that ART may cooperate with Doxorubicin to increase killing of tumor cells. To address this, Jurkat and CEM cells were treated with a combination of ART and Doxorubicin at different doses. Consistently, treatment of Jurkat and CEM cells with either ART or Doxorubicin alone resulted in apoptotic cell death in a dose dependent manner. As expected, treatment of these cells with a combination of ART and Doxorubicin led to a synergistic (not only additive) increase in apoptotic cell death. As shown in Fig. 5A, treatment of Jurkat cells with 25 or 50 ng/ml of Doxorubicin or 2 or 4 µg/ml of ART alone led to about 23 to 28% and 7 to 12% apoptotic death, respectively, whereas, a combination of both drugs resulted in 44 to 87% apoptosis. The synergistic effect of the two drugs was even more significant when the drugs were given to CEM cells. As shown in Fig. 5B, treatment of CEM cells with ART or Doxorubicin alone led to only 7 to 13% and 3 to 4% apoptosis, respectively. However, a combination of both drugs resulted in more than 60% apoptotic cell death. Thus, ART can synergize with Doxorubicin to enhance the efficacy of killing of tumor cells.

## Discussion

We have previously shown that ART inhibits angiogenesis in mice xenografted with Kaposi's sarcoma [14] and trigger apoptotic cell death in various tumor cell lines *in vitro* [19], [20]. Although the cytotoxicity of ART for tumor cells was found a decade ago [15], the molecular mechanisms by which ART exerts its anti-tumor activity have not been elucidated. In this study, using leukemic T cells as a model system, we show that ART induces malignant T cells to undergo apoptosis through the mitochondria pathway. This is demonstrated by inducing the release of cytochrome c from the mitochondria upon ART treatment and followed by activation of caspase-9, the main caspase involved in the intrinsic pathway. In contrast, no activation of caspase-8, the main caspase for the extrinsic pathway, was seen. Furthermore, cells deficient of either the death adapter molecule FADD or caspase-8 were at least as sensitive to ART as the parental cells. Further investigation of the molecular mechanisms by which ART triggers apoptosis revealed that ART induces the intrinsic death pathway by generation of ROS. This is confirmed by the fact that the antioxidant NAC could completely block ROS generation and, consequently, inhibited ART-induced apoptosis.

Resistance to apoptosis may be a key factor of tumor cell survival. A major obstacle for successful cancer treatment often is the development of drug resistance in cancer cells. Therefore, there is an urgent need for novel drugs with improved efficacy against tumor cells and with less toxicity on normal cells. ART shows efficacy against multidrug-resistant *Plasmodium* strains and is surprisingly well tolerated [9], [11]. Thus, ART may be a candidate for use in cancer therapy. As an example, we demonstrate here that ART can overcome apoptosis resistance of Doxorubicin in a resistant cell line. The anticancer drug Doxorubicin (Adriamycin) belongs to the anthracycline-based DNA intercalators. It is well accepted that the anticancer activity of Doxorubicin is caused by inhibition of DNA polymerases, DNA topoisomerase II, and DNA methyltransferase, resulting in induction of apoptosis [31]–[33]. We show that ART induces apoptosis in leukemic T cells *via* generation of ROS, a mechanism different from Doxorubicin. Therefore, ART can induce the Doxorubicin-resistant CEM cells to undergo apoptosis. We also observed that among the four cell lines tested, the acute lymphoblastic leukemia cell lines CEM and Molt-4 were shown to be approximately 5- to10-fold more susceptible to ART treatment than the acute leukemia T cell line Jurkat and the T lymphoma cell line Hut78. Such difference might be due to different expression levels of anti- or/and pro-apoptotic proteins in different types of tumors. In addition, different types of tumors may express different amounts of multi-drug transporter proteins on their plasma membrane which may also account for such differences.

Besides the DNA intercalating function, Doxorubicin has been also proposed to induce oxidative stress in colon tumor cells [34] and cardiac cells [35]. However, we did not detect any ROS generation by Doxorubicin at the doses that induce apoptosis in leukemic T cells. Also, in all leukemic T cell lines tested the Doxorubicin-induced apoptosis could not be blocked by the antioxidant NAC indicating that ROS is not the cause of Doxorubicin-induced apoptotic cell death in these cells. Our result is supported by an early study showing that a low dose of Doxorubicin, at which it could induce ROS generation in Jurkat T cells, led to necrosis, whereas a high dose of Doxorubicin causes apoptosis but no ROS generation [36].

It has been reported that ART and Doxorubicin had synergistic effects on killing of *Plasmodium falciparum*, although the mechanistic aspects have not been addressed [37]. Interestingly, we also found that ART can synergize with Doxorubicin to enhance apoptotic cell death in leukemic T cells. This synergistic effect can be largely explained by the fact that ART and Doxorubicin use different killing mechanisms. DNA intercalating agents, such as Amsacrine, Actinomycin, Mitoxantrone, and Doxorubicin, have been employed as anticancer drugs and are routinely used in the clinic as chemotherapeutic agents [38]. We assume that ART may also cooperate with other DNA intercalating anticancer drugs under the same principle. Thus, our studies raise the possibility to develop ART in combination with other established anticancer drugs which induce apoptosis through the pathways or mechanisms different from ART.
